# Childhood ADHD Symptoms: Association with Parental Social Networks and Mental Health Service Use during Adolescence

**DOI:** 10.3390/ijerph120911893

**Published:** 2015-09-22

**Authors:** Regina Bussing, Johanna Meyer, Bonnie T. Zima, Dana M. Mason, Faye A. Gary, Cynthia Wilson Garvan

**Affiliations:** 1 Department of Psychiatry, University of Florida, Box 100256, Gainesville, FL 32610-0256, USA; E-Mail: dmason@ufl.edu; 2 School of Psychology, University of Wollongong, Northfields Ave., Wollongong NSW 2522, Australia; E-Mail: jmm730@uowmail.edu.au; 3 UCLA-Semel Institute for Neuroscience and Human Behavior, 10920 Wilshire Blvd. Suite 300, Los Angeles, CA 90024, USA; E-Mail: bzima@mednet.ucla.edu; 4 Frances Payne Bolton School of Nursing, Case Western Reserve University, 2120 Cornell Road, Cleveland, OH 44106, USA; E-Mail: fxg21@case.edu; 5 Office for Research Affairs, College of Nursing, University of Florida, Gainesville, FL 32610, USA; E-Mail: cgarvan@ufl.edu

**Keywords:** attention-deficit/hyperactivity disorder, community sample, social support networks, caregiver strain, mental health services utilization

## Abstract

*Objective*: This study examines the associations of childhood attention-deficit/hyperactivity disorder (ADHD) risk status with subsequent parental social network characteristics and caregiver strain in adolescence; and examines predictors of adolescent mental health service use. *Methods*: Baseline ADHD screening identified children at high risk (n = 207) and low risk (n = 167) for ADHD. At eight-year follow-up, parents reported their social network characteristics, caregiver strain, adolescents’ psychopathology and mental health service utilization, whereas adolescents self-reported their emotional status and ADHD stigma perceptions. Analyses were conducted using ANOVAs and nested logistic regression modeling. *Results*: Parents of youth with childhood ADHD reported support networks consisting of fewer spouses but more healthcare professionals, and lower levels of support than control parents. Caregiver strain increased with adolescent age and psychopathology. Increased parental network support, youth ADHD symptoms, and caregiver strain, but lower youth stigma perceptions were independently associated with increased service use. *Conclusions*: Raising children with ADHD appears to significantly impact parental social network experiences. Reduced spousal support and overall lower network support levels may contribute to high caregiver strain commonly reported among parents of ADHD youth. Parental social network experiences influence adolescent ADHD service use. With advances in social networking technology, further research is needed to elucidate ways to enhance caregiver support during ADHD care.

## 1. Introduction

Attention-deficit/hyperactivity disorder (ADHD) is a chronic neurodevelopmental disorder with significant public health implications, because it is (1) common, affecting more than 1 in 20 children [1]; (2) associated with significant adverse outcomes for youth [2,3] and their family members [4]; and (3) treatable with appropriate diagnosis and intervention [5,6]. Although national guidelines to improve the quality of care were developed [7] and significant progress has been made to standardize assessment procedures and treatment [8,9], access to ADHD care remains poor and racial and sociodemographic disparities in care persist [10,11]. 

Parental social networks may be one important contributor to such disparities, influencing all stages of the help-seeking process and illness career trajectories for pediatric chronic health conditions [12]. Most influential conceptual models of access to health care assume that health care utilization is influenced by need (e.g., disorder severity and functional impairment), along with other barriers and facilitators of access [13]. Of these models, the family network-based model is well-suited to capture the complex processes influencing whether mental disorder in youth is detected, considered potentially treatable, and what types of interventions may be called for. Network-based models shift the focus from individual rational decision-making to socially constructed decision-making patterns [14]. Networks are thought to provide the interaction through which affected families learn about, understand and resolve difficulties [12]. Previous research confirmed connections between social networks and children’s access to mental health care. A study examining the social networks of parents with children with ADHD found that African-American parents reported having smaller social networks, but more frequent contact with their network members and higher levels of support than Caucasian parents, with instrumental support inversely related to the odds of children receiving ADHD treatment [15]. Social network members may also buffer parents’ stress and improve their health outcomes [16], and depending on transmitted messages and beliefs, networks may impede or facilitate access to treatment [17,18,19,20]. 

Another important predictor of child mental health service utilization is parental stress or perceived burden of the child’s mental disorder. High amounts of parental stress, common in parents of children with ADHD [21,22,23,24], are associated with increased utilization of general medical [25,26,27,28,29,30] and mental health services [10,12,15,31]. Extensive research has demonstrated that social support buffers stress in various populations, including parents of children with ADHD [32,33,34,35,36]. Therefore, while parental stress may increase the likelihood of a child receiving treatment for ADHD, strong social support may reduce mental health service utilization by buffering this stress. 

Furthermore, the relationship between social networks, caregiver stress and youth health care utilization has been examined for parents of young children with ADHD [15,34,35], but not yet for parents of affected adolescents. This is an important limitation in our understanding of adolescents’ access to ADHD care, because teenagers’ developmental maturation and desire for self-determination likely influence whether and how they access mental health services. Adolescents become increasingly sensitive to peer opinions and perceived stigma of mental disorders, including ADHD [37]. There may be other age-related changes, such as parental stress increasing over time as defiance and delinquency associated with ADHD wears on parents [38], or possible loss of social network support due to the repeated stigmatizing disturbances caused by the child’s disorder [39]. In a representative national study of social reactions to children with mental health problems, public stigmatizing reactions were reported to be higher for adolescents than younger children, possibly producing higher courtesy stigma burden for the parents of teenagers with mental disorders [40]. Yet, absent of new study findings, one may also hypothesize that the influence of parental network support and stress experiences on access to care diminishes during adolescence because teens increasingly exert their own treatment preferences [37]. To address this knowledge gap, this study has three related objectives: 

(1) To compare social network characteristics of parents of adolescents with and without a history of childhood ADHD. We hypothesize that childhood ADHD is associated with smaller parent network size and less emotional and instrumental support as the children reach adolescence.

(2) To identify predictors of caregiver stress in in parents of adolescents. We hypothesize that adolescent age and psychopathology are positively associated with caregiver stress experiences; and conversely, that larger network size and greater support are associated with reduced caregiver stress.

(3) To ascertain whether parental network composition and stress predict mental health service utilization for adolescents, after controlling for mental health need and adolescents’ ADHD stigma perceptions. No hypothesis was advanced, due to the absence of existing research informing this question.

## 2. Experimental Section 

### 2.1. Participants and Procedures

This research uses observational data from the study “ADHD: Detection and Service Use” [15]. A random sample of children, oversampling girls by a factor of two, was derived from public school records of all 12,009 kindergarten through fifth graders from a North Florida school district. Baseline screening of 1615 children occurred in 1998 and 1999, consisting of comprehensive parental telephone interviews, including administration of the Swanson-Nolan-and Pelham-IV (SNAP-IV), a standardized ADHD screening measure, plus eliciting detailed history of potential behavioral problems and mental health service use history, followed by, if parental consent was provided, teacher completion of the SNAP-IV and a functional screener. Teacher ratings were completed on paper and handled through mailings. Screenings identified 476 (29.5%) children as high risk for ADHD because they were either already diagnosed or treated for ADHD, were suspected by parents or school as having ADHD, or elicited parent or school concerns about behavioral problems plus their standardized parent ADHD screening scores were at least 1.5 standard deviations above the norm. Of these high-risk children, 207 remained eligible for inclusion in the social network study and participated on average eight years after the initial screening, between 2005 and 2008. Of 1139 peers classified as low risk at baseline, we matched 167 youths to the high-risk participants by gender, race, poverty status and age to serve as comparison group. Among the total sample of 374 adolescents included in the social network study, the average age was 15.4 years old (range 11 to 20 years, SD = 1.8), 57% (n = 213) were female, 37% (n = 137) were African-American, and 52% (n = 196) received lunch subsidies, an indicator of poverty. Of their 374 caregivers 37% (n = 137) were African American, and 52% (n = 196) were of low socioeconomic status (Hollingshead IV or V). The majority was biological mothers, but caregivers also included step-parents, grandparents, and other types of legal guardians (henceforth referred to as caregivers or parents). Individual interviews for the social network study were conducted by trained research assistants with adolescents and parents in homes, community locations, or at our research center. The study was approved by the University Institutional Review Board and the school district research office. Informed consent (parents and young adults) or child assent (adolescents) was obtained from all subjects, and a stipend of $40 (parents) or $30 (adolescents) was given for their participation.

### 2.2. Study Variable Construction 

#### 2.2.1. Sociodemographic Characteristics 

Sociodemographic information about child age, gender, race, and subsidized status was obtained from school registration records and verified during interviews. 

#### 2.2.2. Adolescent Clinical Characteristics

##### Psychopathology

Symptoms of common mental health problems were assessed by parent report using the Vanderbilt ADHD Diagnostic Parent Rating Scale (VADPRS). Parents completed the 47-item VADPRS, used to assess symptoms of ADHD, oppositional defiant disorder (ODD), conduct disorder (CD), anxiety and depression. The VADPRS uses a 4-point scale (0 = never, 3 = very often), and the scores for each disorder corresponds to the total sum. The reliability, factor structure, and concurrent validity have been found to be acceptable and consistent with the DSM-IV and other accepted measures of ADHD [41]. 

##### Impairment

To assess child functional impairment, parents and adolescents completed the Columbia Impairment Scale (CIS), a 13-item survey which yields an overall impairment score that can range from 0 to 52, with scores of 15 or greater indicating definite impairment [42]. The CIS has been reported to have high internal consistency and test-retest reliability and correlations with clinician and parent ratings of child impairment were both highly significant, supporting the construct validity of the measure [42].

##### Emotional and Behavioral Functioning

Adolescents completed the Behavior Assessment System for Children Self Report of Personality (BASC-SRP), a 186-item instrument using a true/false answer format which yields an overall summary score of emotional and behavioral functioning, the Emotional Symptom Index (ESI) [43]. The BASC-SRP was normed using 9861 U.S. children, has been used in diverse populations, and is known to have adequate reliability and validity [43]. Scores are converted to *T* scores, with higher ratings indicative of greater impairment [43].

#### 2.2.3. Adolescent Perceived Stigma

Adolescents completed the 26-item ADHD Stigma Questionnaire (ASQ) to assess public stigma perceptions related to ADHD [44]. Adolescents respond to statements, such as “People with ADHD work hard to keep it a secret” on a 4-point scale (1 = strongly disagree, 2 = disagree, 3 = agree, 4 = strongly agree). The measure yields an overall stigma score (range: 1–4, higher score = more perceived stigma). The ASQ has been shown to have acceptable psychometric properties in a diverse community sample of adolescents, including high internal consistency (α = 0.93), good test-retest reliability (2-week intraclass correlation coefficient = 0.71), and support for convergent and divergent validity [44].

#### 2.2.4. Parental Social Network Characteristics

The Norbeck Social Support Questionnaire (NSSQ) was adapted to assess parents’ perceived level of network support specific to their caregiver role, defined as persons whom caregivers talk to and depend on when they have concerns about their child’s health, behavior, or emotions [45]. The NSSQ measures social network size, network composition, contact frequency, length of relationship, geographical distance from network members, and the amount of perceived support. Perceived support from each network member is measured by three variables: affective (e.g., feeling liked or loved), affirmational (e.g., ability to confide, agreement with parenting decisions), and instrumental support (e.g., help with babysitting or other parenting responsibilities if parent confined to bed, or help with transportation if parent needs a ride to the doctor’s office). Each of the three support variables represents the sum of two items measured on a scale from 0 (“not at all”) to 4 (“a great deal”), yielding scores that can range from 0 to 8. Test-retest reliability (ranging from 0.85 to 0.92), internal consistency (ranging from 0.88 to 0.96), and concurrent validity of the NSSQ have been established in both a sample of graduate and undergraduate nursing students [45], and a sample of hospital staff workers [46]. To avoid potential confounding between network size and amount of support, this study utilized average support rates, therefore controlling for differences in network size [47]. The respondents in this study consisted almost entirely of mothers (98%).

#### 2.2.5. Caregiver Stress

The impact of caring for a child with emotional or behavioral problems on the parent was assessed using the Caregiver Strain Questionnaire (CGSQ), a 21-item adult self-report instrument [48]. Caregivers rated how much of a problem each situation or feeling was in the past six months as a result of his or her child’s problems. Examples of the areas addressed in the questionnaire are family disruption, time demands, financial strain, worry, guilt, and embarrassment. Responses are scored on a five-point scale from “not at all” to “very much” a problem. The questionnaire measures three caregiver stress dimensions: objective (eleven items), internalized subjective (six items), and externalized subjective (four items) strain. The CGSQ has been shown to be an effective measure of caregiver stress in both African American and Caucasian families [49]. The measure demonstrated acceptable internal consistency in the current study, with an alpha coefficient of 0.96 for the entire scale. 

#### 2.2.6. Mental Health Service Use

Receipt of mental health treatment services was assessed using the Child and Adolescent Services Assessment (CASA) [50], a parent-report measure that inquires about usage of mental health services from 33 treatment settings, including inpatient, outpatient, and informal care. In cases when a mental health service was used, detailed information about service use in the past 12 months, such as number of sessions, type of services, and prescribed medication type, dose, and frequency, was collected. Validity studies have shown good to excellent agreement between CASA parent reports and medical records for receiving outpatient services [51,52] and also excellent agreement on details of the child’s medication regimens [52]. Adolescents were identified as receiving mental health treatment in the past 12 months when their parent reported any inpatient or outpatient mental health care during 12 months before the date of the interview. 

### 2.3. Statistical Analyses

**Figure 1:**
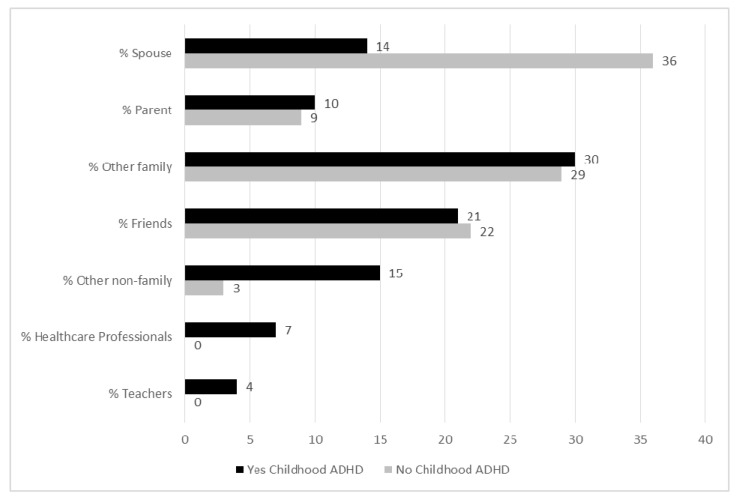
Percent of network composition by support type, by ADHD status.

Data were checked for implausible values, outliers, distributional form and missing information. Source of support was categorized into five “informal” types (spouse, parent, other family, friends, and other) and two “formal” types (healthcare and teacher). To describe networks of the parent participants reported in Table 1, summary statistics were computed for the ordinal variables of network structure (contact frequency, relationship length, geographical distance, and emotional closeness) and categorical variables of network support (affective, affirmative, instrumental). Since multiple sources in each of the informal and formal network types are possible, it was necessary to compute summary statistics within participants before computing overall summary statistics. For example, if a participant listed six friends in their network structure, the within median/mean were computed for that individual participant and subsequently used to calculate the overall sample median/mean. Due to the variety of patterns possible for network structures, statistical testing was not performed for the summary statistics given in Table 1. It was, however, possible to statistically compare the composition of network structure (*i.e.*, percent of support types in each structure). For comparison of this composition (depicted in Figure 1), chi-square testing was performed. For the chi-square testing, teacher and healthcare professional were collapsed into a single category of “formal care.” For comparisons in Table 2 of network size, support type (percent composition of network by each informal and formal type), network structure, and network support by the explanatory variables of race, poverty, and childhood ADHD risk status, individual-level summary statistics of affective, affirmative, instrumental support variables were examined using *t*-tests, and Wilcoxon rank sum tests were performed for ordinal variables of network structure (contact frequency, relationship length, geographical distance, and emotional closeness). Spearman correlational testing was performed to examine the relationship of network variables (network size, affective support, affirmative support, and instrumental support) with global caregiver strain. To examine the dependent variable of global caregiver strain and the network variables in the correlational analysis after adjusting for the explanatory predictors (child age, gender, poverty, race, symptoms of ADHD and of anxiety/depression), a multiple regression model was constructed. Comorbid ODD was considered as an additional predictor in the multiple regression model, but the extent of multicollinearity with ADHD was unacceptable (*r* > 0.70). For the series of logistic models presented in Table 3, logistic modeling was used to examine three nested models that predicted mental health treatment use in the past 12 months. Model 1 included network characteristics as predictor variables (size, affective support, affirmative support, and instrumental support), Model 2 added parental perspectives of child need (anxiety/depression, ADHD, functioning) and global caregiver strain. Model 3 added the adolescent perceptions of emotional and behavioral functioning, impairment, and stigma. Significant improvement in model fit for the nested logistic regressions was tested using goodness of fit analysis. A level of significance of 0.05 was specified for all significance testing. SAS version 9.3 software (Cary, N.C.) was used for all descriptive and inferential analyses.

## 3. Results and Discussion

### 3.1. Parent Network Structure and Support Levels by Type of Support Source

Family support sources were the most commonly reported, with 61% (n = 230), 44% (n = 165), and 60% (n = 227) of the caregivers listing spouses, their own parents or other family members, respectively. Friends represented 52% (n = 195) of the caregiver supports, and formal network supporters like healthcare providers or teachers, were only listed as a support source by 14% (n = 54) and 4% (n = 15) of the caregivers. Not surprisingly, the mean contact frequency, relationship length and emotional closeness were greater for informal than formal network supporters, and geographical distance was least for spouses. Informal network supports also provided higher levels of affective/affirmative and instrumental support than informal sources, with the most distinct differences in instrumental support experiences. Details on network structure and support levels are summarized in Table 1.

**Table 1:** Parent network structure and support levels by type of support source.

	Informal Network Support Mean (SD)				Formal Network Support Mean (SD)		
	Spouse	Parent	Other Family	Friends	Other	Healthcare	Teacher
	N ^a^ = 230	N ^a^ = 196	N ^a^ = 420	N ^a^ = 356	N ^a^ = 154	N ^a^ = 67	N ^a^ = 18
	n ^b^ = 230	n ^b^ = 165	n ^b^ = 227	n ^b^ = 195	n ^b^ = 105	n ^b^ = 54	n ^b^ = 15
Network Structure							
Contact Frequency ^c^	4.9 (0.5)	4.5 (0.7)	4.4 (0.7)	4.4 (0.7)	4.4 (0.7)	2.6 (0.7)	3.6 (0.7)
Relation Length ^d^	4.9 (0.3)	5.0 (0.2)	5.0 (0.1)	4.8 (0.5)	4.5 (0.9)	3.6 (1.4)	2.8 (1.3)
Geographical Distance ^e^	1.6 (1.3)	4.7 (1.6)	4.6 (1.6)	4.3 (1.0)	4.2 (0.8)	4.1 (0.3)	4.2 (0.6)
Emotional Closeness ^f^	1.2 (0.4)	1.2 (0.5)	1.1 (0.3)	1.2 (0.4)	1.5 (0.6)	2.0 (0.7)	2.1 (0.4)
Network Support							
Affective ^g^	3.5 (0.8)	3.6 (0.7)	3.6 (0.5)	3.6 (0.5)	3.4 (0.7)	3.0 (0.8)	2.9 (1.0)
Affirmative ^g^	3.5 (0.6)	3.6 (0.7)	3.6 (0.5)	3.6 (0.5)	3.4 (0.7)	3.0 (0.8)	2.9 (1.0)
Instrumental ^g^	3.6 (0.8)	2.8 (1.4)	2.8 (1.2)	2.7 (1.1)	1.9 (1.3)	0.5 (0.8)	0.8 (1.3)

^a^ Total number of specific support types; ^b^ Number of parent respondents listing such support type; ^c^ Frequency of contact with support person from 1 (once a year or less) through 5 (daily); ^d^ Length of relationship from 1 (less than 6 months) to 5 (5 years or more); ^e^ Geographical distance from support person from 1 (same household) to 7 (out of state); ^f^ Emotional closeness from 1 (very close) to 3 (not very close); ^g^ Each represents the average of two questions, scored from 0 (not at all) to 4 (a great deal).

### 3.2. Parent Network Support Type Composition by ADHD Status

As shown in Figure 1, support networks of parents were significantly different by ADHD status (*p* < 0.0001). The support networks of parents whose teen had a history of childhood ADHD were composed of fewer spouses compared to parents of teens without such history (14% *versus* 36%). Conversely, support networks of parents whose teen had a childhood ADHD history indicated more distal network support than parents of teens without such history, including more teachers (4% *versus* 0%), healthcare professionals (7% *versus* 0%), and other non-family connections (15% *versus* 3%). 

### 3.3. Parent Network Characteristics by ADHD Status, Poverty and Race

As summarized in Table 2, the average number of network supporters was 3.9 (SD 2.2) and did not differ by childhood ADHD status, but did significantly vary by poverty status and race. Poor or African-American caregivers had smaller networks than non-poor (*p* < 0.0001) or Caucasian peers (*p* < 0.0001). In addition, the average contact frequency was high, 4.5 (SD 0.6), indicating near daily contacts and did not vary by childhood ADHD status, but by poverty status and race/ethnicity. Poor or African American caregivers had more frequent contact than non-poor (*p* < 0.0001) or Caucasian peers (*p* < 0.0001).

Receipt of affective and affirmative support averaged 3.4 (SD 0.8) and 3.3 (SD 0.7), respectively, and was lower for caregivers of youth with childhood ADHD status than those of caregivers without (*p* < 0.0001 and *p* < 0.01, respectively), but did not vary by poverty status or race. Average instrumental support was only 2.7 (SD 1.0) and varied by all three conditions, with caregivers of youth with childhood ADHD status (*p* < 0.0001), non-poor (*p* < 0.05) and Caucasian caregivers (*p* < 0.0005) reporting lower instrumental supports than caregiver peers of the opposite classification.

**Table 2:** Parent network characteristics by childhood ADHD risk status and sociodemographic characteristics.

Overall Mean (SD)		By ADHD Risk Status Mean (SD)			By Poverty ^a^ Mean (SD)			By Race ^b^ Mean (SD)		
		Low N = 167	High N = 207	*p*	Non-Poor N = 178	Poor N = 196	*p*	C N = 237	AA N = 137	*p*
Network Size	3.9 (2.2)	3.7 (2.2)	3.9 (2.3)	0.57	4.4 (2.3)	3.4 (2.1)	<0.0001	4.2 (2.3)	3.3 (2.1)	<0.001
Network Structure										
Contact Frequency ^c^	4.5 (0.6)	4.5 (0.6)	4.5 (0.7)	0.34	4.4 (0.7)	4.6 (0.6)	<0.0001	4.4 (0.7)	4.6 (0.6)	<0.0001
Relation Length ^d^	4.9 (0.4)	5.0 (0.2)	4.9 (0.5)	<0.01	4.9 (0.3)	4.9 (0.4)	0.42	4.9 (0.4)	4.9 (0.3)	0.81
Closeness ^e^	1.2 (0.4)	1.1 (0.3)	1.2 (0.4)	<0.05	1.2 (0.4)	1.1 (0.4)	0.06	1.2 (0.4)	1.1 (0.3)	<0.05
Geographic Distance ^f^	4.0 (1.2)	4.0 (1.3)	4.1 (1.1)	0.40	4.2 (1.2)	3.8 (1.2)	<0.01	4.1 (1.2)	3.8 (1.2)	<0.05
Network Support										
Affective ^g^	3.4 (0.8)	3.6 (0.7)	3.3 (0.8)	<0.0001	3.5 (0.6)	3.4 (0.9)	0.94	3.4 (0.6)	3.5 (1.0)	0.25
Affirmative ^g^	3.3 (0.7)	3.4 (0.7)	3.2 (0.8)	<0.01	3.3 (0.6)	3.3 (0.9)	0.60	3.3 (0.6)	3.3 (1.0)	0.33
Instrumental ^g^	2.7 (1.0)	2.9 (0.9)	2.4 (1.0)	<0.0001	2.6 (0.9)	2.8 (1.1)	<0.05	2.5 (0.9)	2.9 (1.1)	<0.001

^a^ Poverty status assigned according to subsidized school lunch status (non-poor means no subsidy; poor means free or reduced lunch); ^b^ Race distinguishes C = Caucasian, AA = African American; ^c^ Frequency of contact with support person from 1 (once a year or less) through 5 (daily); ^d^ Length of relationship from 1 (less than 6 months) to 5 (5 years or more); ^e^ Closeness of relationship from 1 (very close) to 3 (not very close); ^f^ Distance from support person from 1 (same household) to 7 (out of state); ^g^ Each represents the average of two questions, scored from 0 (not at all) to 4 (a great deal).

### 3.4. Predictors of Caregiver Stress

Global caregiver strain was independently associated with adolescents’ age (beta coefficient 0.17; *p* < 0.0005) and ADHD and anxiety/depression symptom scores (beta coefficients 1.47; *p* < 0.0001 and 1.38; *p* < 0.0001; respectively); and at a trend level, with caregiver’s affective support (*i.e.*, feeling liked or loved; beta coefficient 0.39; *p* < 0.10). No independent relationships were found between global caregiver strain and the remaining demographic (*i.e.*, gender, race, poverty) or network characteristics (*i.e.*, network size, affirmational or instrumental support).

### 3.5. Predicting Adolescent Mental Health Service Utilization

Network size and affective support (*i.e.*, feeling liked or loved) were not associated with adolescents’ mental health service use. However, parental receipt of network affirmational support (*i.e.*, having a confidant and someone who agrees with parenting decisions) was associated with increased odds of adolescents’ mental health service use in Model 1 (OR 1.92, 95% CI 1.03–3.57), and remained an independent predictor after accounting for parental perspectives of adolescents’ psychopathology and their own caregiver strain experiences (Model 2, affirmation support OR 2.50, 95% CI 1.23–5.06), and after controlling for adolescents’ need (*i.e.*, their emotional and behavioral functioning and impairment status, ESI and CIS, respectively) and ADHD stigma perceptions (Model 3; affirmation support OR 2.60, 95% CI 1.23–5.51). Parental receipt of instrumental support (*i.e.*, help with parenting responsibilities) was associated with lower odds of adolescents’ mental health service use in Model 1 (OR 0.69, 95% CI 0.52–0.92), but did not retain significance after accounting for parental and adolescent perspectives in Models 2 and 3, respectively. 

As shown in Table 3, goodness of fit estimates increased significantly with each subsequent nested model. The best fit was obtained with Model 3, which contained network characteristics, parent and adolescent perspectives. In Model 3, in addition to affirmation support, adolescents’ mental health service utilization was independently associated with parental reports of adolescents’ ADHD symptoms (OR 1.97, 95% CI 1.18–3.29) and of caregiver strain (OR 1.29, 95% CI 1.03–1.60), and with adolescents’ ADHD stigma perceptions (OR 0.30, 95% CI 0.15–0.59).

The findings of this study confirm the hypothesis that childhood ADHD adversely impacts the social network composition of their caregivers. Even though social networks did not differ in size, parents who had raised children with ADHD received less spousal support than peers with unaffected children, and they relied on more distal or formal supporters, such as friends, teachers or healthcare professionals. Our findings are consistent with prior studies reporting negative associations between childhood ADHD and parental relationship stability [53,54] that were limited by small sample sizes and convenience sampling methods. More recently, a large Danish registry-based study confirmed that childhood ADHD significantly reduced parental relationship stability over a 10-year time period [55]. Despite maintaining similar network sizes as parents of unaffected children and the addition of new types of social support, parents who raised children with ADHD experienced lower levels of support in all domains (affective, affirmative and instrumental) and felt overall less close to their network members. No previous studies could be identified that addressed this particular finding. Nevertheless, results from the National Stigma Study-Children suggest possible explanations for ADHD-affected parents’ experiences of lowered support include preferences for social distance from those perceived to have mental problems [40]. We therefore propose that as a consequence of the well-described phenomenon of courtesy stigma [56,57], parents of a child with ADHD are likely to experience similar social distancing that may result in lower social support levels.

**Table 3:** Predictors of Mental Health Treatment Use in Past 12 Months.

	Model 1 (Network)		Model 2 (Network, Parent Perspectives)		Model 3 (Network, Parent Perspectives, Adolescent Perspectives)	
	OR	[95% CI]	OR	[95% CI]	OR	[95% CI]
Network Characteristics						
Network size	1.07	[0.97, 1.19]	1.04	[0.92, 1.17]	1.05	[0.92, 1.19]
Affective support	0.68	[0.36, 1.28]	0.85	[0.41, 1.75]	0.73	[0.34, 1.60]
Affirmation support	**1.92**	**[1.03, 3.57]**	**2.50**	**[1.23, 5.06]**	**2.60**	**[1.23, 5.51]**
Instrumental support	**0.69**	**[0.52, 0.92]**	0.93	[0.66, 1.30]	0.91	[0.63, 1.32]
Parent Perspectives						
Anxiety/Depression ^a^			**2.12**	**[1.11, 4.08]**	1.62	[0.78, 3.29]
ADHD ^a^			**1.88**	**[1.18, 3.01]**	**1.97**	**[1.18, 3.29]**
CIS			1.02	[0.96, 1.08]	0.98	[0.92, 1.05]
Caregiver strain			**1.23**	**[1.02, 1.50]**	**1.29**	**[1.03, 1.60]**
Adolescent Perspectives						
ESI					1.02	[0.97, 1.06]
CIS					1.05	[0.99, 1.11]
ADHD Stigma					**0.30**	**[0.15, 0.59]**
2 LOG L ^b^	419.2		347.8, (71.397,4), *p* < 0.0001 ^c^		307.1, (40.625,3), *p* < 0.0001 ^c^	

*Notes:* OR = odds ratio; 95% CI = 95% confidence interval; CIS = Columbia Impairment Scale; **Bolded OR and 95% CI** = *p* < 0.05. ^a^ average rating index scores from Vanderbilt ADHD Diagnostic Parent Rating Scale; **^b^** Significant improvement in model fit was tested using goodness of fit analysis (*i.e.*, computation of chi-square statistics based on differences of—2 LOG L between models and the corresponding difference of model degrees of freedom); ^c^ Model fit significantly improved over previous model.

As expected, caregiver stress increased with youth age and the degree of adolescent psychopathology, as indicated by ADHD and anxiety/depression symptom levels. High levels of caregiver burden have been reported in other studies of ADHD patients transitioning into adolescence and adulthood, with burden being a function of the affected young person’s unmet need, especially depression/anxiety and behavioral problems [58]. However, contrary to our hypothesis, network support did not appear to significantly reduce caregiver stress. Only at a trend level was affective support (*i.e.*, the experience of feeling liked or loved by network members) associated with lowered strain scores. Total network size, affirmational and instrumental support were also not related to parent stress. Our findings are in contrast to other studies reporting that greater social support is related to reduced stress [33]; however, the discrepancy may be due to measurement differences or reflect an overly simplistic measurement concept of the relationships between social support and stress buffering. As has been proposed by Cohen, social support is only effective in reducing the effects of stressful events if the form of support matches the demands of the stressful experience [59], and the current study did not attempt to identify specific support needs contributing to caregiver stress.

Furthermore, findings suggest that affirmational support from the parents’ social network, caregiver stress, youth ADHD symptom level, and reduced adolescent perceived stigma were predictive of mental health service utilization among teens. To our knowledge, this is the first study to specifically address the question of how parental network composition and stress play a role in mental health service utilization for adolescents, even after controlling for their mental health needs and teens’ ADHD stigma perceptions. Interestingly, whereas earlier research found that caregivers’ instrumental support, (*i.e.*, getting help with parenting responsibilities) lowered the odds of mental health service use of children [15], such type of parental support no longer was associated with mental health service use in adolescents. In contrast, caregivers’ affirmational support (*i.e*., having a confidant that agrees with the caregiver’s parenting decisions) increased the odds of mental health service use of adolescents. We speculate that this observed relationship may reflect the parent’s active assembly of network supporters who view mental health treatment as beneficial (including non-family members, teachers and health professionals) and who do not impose courtesy stigma on parents for seeking mental health treatment for their adolescent. Considering the study’s findings regarding spousal support, we note that previous research has documented the reluctance of fathers to accept medical frameworks for understanding ADHD [60]. We also suggest that further research should examine the potential role of conflicting parental ADHD explanatory models in causing caregiver stress and destabilizing the parents’ relationship. Furthermore, the findings of the study indicate that adolescents’ stigma perceptions surrounding ADHD, but not their own perceptions of emotional adjustment or functional impairment, play a role in their access to mental health care. Together these findings suggest that quality improvement interventions aimed at improving mental health care access for adolescents with ADHD should seek to improve affirmational parental support as well as reduce caregiver stress and perceived ADHD stigma among teens.

Study findings should be interpreted in the context of several limitations. The study was limited to one area of North Florida, and even though participants were identified through representative sampling and included diverse respondents, findings may not generalize to other regions, or respondents of racial/ethnic groups not represented in this regional sample. Furthermore, the designation of childhood ADHD risk status was based on screening results, not on confirmatory diagnostic assessments. If stricter diagnostic criteria had been applied, differences to the control group might have been even more pronounced. Finally, the instrument used to assess social network composition and support was not tooled to identify whether networking included internet-based social networking methods. Over the past decade Social Networking Sites (SNS) such as Facebook, Twitter, LinkedIn, and MySpace have greatly increased in popularity, with the number of American adults who use SNSs nearly doubling from 26% in 2008 to 47% in 2010 [61]. However, at the time the current study was designed SNS had not risen to popularity yet and the NSSQ was the only available suitable network assessment tool; we assert that the results obtained still make a novel contribution to our understanding of caregiver networks supports for this common neurodevelopmental disorder. 

## 4. Conclusions 

Study findings suggest that raising children with ADHD significantly impacts parental social network experiences. Reduced spousal support and overall lower network support experiences may contribute to high caregiver stress commonly reported among parents of ADHD youth. Parental social network experiences appear to influence adolescent mental health service use. With advances in social networking technology, a better understanding is needed on how to integrate SNS use into management models for chronic conditions like ADHD. For example, SNS users reportedly have more diverse social networks, more social ties, and interact more with members of their social network in both offline and online settings than non-users [61,62]. Along with these social effects, SNS usage is associated with increased well-being, self-esteem, and life satisfaction [63,64,65,66], which could decrease the perceived burden of caring for a child with ADHD and open avenues to enhance family and self-management. Further research is needed to elucidate ways to enhance caregiver support during ADHD treatment that more closely aligns with youth development and capitalizes on technologic advances that promote social networking.
